# Diversity and phylogenetic analyses of bacteria from a shallow-water hydrothermal vent in Milos island (Greece)

**DOI:** 10.3389/fmicb.2013.00184

**Published:** 2013-07-08

**Authors:** Donato Giovannelli, Giuseppe d'Errico, Elena Manini, Michail Yakimov, Costantino Vetriani

**Affiliations:** ^1^Department of Biochemistry and Microbiology, Rutgers UniversityNew Brunswick, NJ, USA; ^2^Institute of Marine and Coastal Sciences, Rutgers UniversityNew Brunswick, NJ, USA; ^3^Institute for Marine Science - ISMAR, National Research Council of Italy - CNRAncona, Italy; ^4^Institute Coastal Marine Environment - IAMC, National Research Council of Italy - CNRMessina, Italy

**Keywords:** bacteria, *Epsilonproteobacteria*, shallow-water hydrothermal vent, Milos, geothermal

## Abstract

Studies of shallow-water hydrothermal vents have been lagging behind their deep-sea counterparts. Hence, the importance of these systems and their contribution to the local and regional diversity and biogeochemistry is unclear. This study analyzes the bacterial community along a transect at the shallow-water hydrothermal vent system of Milos island, Greece. The abundance and biomass of the prokaryotic community is comparable to areas not affected by hydrothermal activity and was, on average, 1.34 × 10^8^ cells g^−1^. The abundance, biomass and diversity of the prokaryotic community increased with the distance from the center of the vent and appeared to be controlled by the temperature gradient rather than the trophic conditions. The retrieved 16S rRNA gene fragments matched sequences from a variety of geothermal environments, although the average similarity was low (94%), revealing previously undiscovered taxa. *Epsilonproteobacteria* constituted the majority of the population along the transect, with an average contribution to the total diversity of 60%. The larger cluster of 16S rRNA gene sequences was related to chemolithoautotrophic *Sulfurovum* spp., an *Epsilonproteobacterium* so far detected only at deep-sea hydrothermal vents. The presence of previously unknown lineages of *Epsilonproteobacteria* could be related to the abundance of organic matter in these systems, which may support alternative metabolic strategies to chemolithoautotrophy. The relative contribution of *Gammaproteobacteria* to the Milos microbial community increased along the transect as the distance from the center of the vent increased. Further attempts to isolate key species from these ecosystems will be critical to shed light on their evolution and ecology.

## Introduction

Microbes are one of the most abundant life forms on Earth, they are ubiquitous, possess a great metabolic plasticity and drive major biogeochemical cycles (Staley and Reysenbach, 2002). Studies carried out in geothermal and extreme environments have shown that life on Earth is far more diverse, widespread, and resistant to extreme conditions than previously thought. Despite the crucial roles of prokaryotes in extreme ecosystems, our understanding of their diversity and ecological relevance in these environments is limited.

Deep-sea hydrothermal vent ecosystems are largely based on chemolithoautotrophic primary production (Jannasch, 1985; Bach et al., 2006; Nakagawa and Takai, 2008). Photoautotrophic contribution to these ecosystems is limited to the sinking of low-quality particulate material from the photic zone (Comita et al., 1984). In contrast, shallow-water hydrothermal vent systems (located at depth <200 m) are largely influenced by photosynthesis (Tarasov et al., 2005). In these environments, chemolithoautotrophy and photoautotrophy occur simultaneously and spatial separation is often influenced by steep thermal and geochemical gradients (Wenzhöfer et al., 2000).

Shallow-water hydrothermal vents are widespread ecosystems that have been previously understudied compared to their deep-sea counterparts (InterRidge vents database, http://www.interridge.org/), despite the fact that these systems were known long before the discovery of the deep-sea vents on the Galapagos Rift in 1977 (Lonsdale, 1977). Because of their proximity to the surface, shallow-water hydrothermal systems are influenced both by geothermally generated reducing power and by light, and can be described as “high energy” environments, where microbial metabolism is fueled by different energy sources (Baross and Hoffman, 1985). According to Dando et al. (1995, 2000), the emission of carbon dioxide from the Milos venting area alone (35 km^2^) could account for 10% of the carbon dioxide emission caused by the venting associated with the Mid-Oceanic Ridges. It has been suggested that the conditions found in shallow-water hydrothermal systems could resemble those in which life originated and where metabolic divergence begun (Nisbet and Fowler, 1996; Nisbet and Sleep, 2001; Martin et al., 2008).

Understanding the microbiology of shallow-water hydrothermal vents is necessary to evaluate how microorganisms influence biogeochemical cycles. The geology and chemistry of the hydrothermal system located in Paleochori Bay, a sandy bay off the South East coast of the island of Milos, was previously investigated by Dando et al. (2000) and Valsami-Jones et al. (2005). In Paleochori Bay, the vents are located in shallow waters, with temperatures ranging from 25 to 119°C (Botz et al., 1996) and extensive gas and fluid seepage. Early microbiological studies based on fingerprinting approaches showed the presence of bacteria associated to the *Cytophaga-Flavobacteria-Bacteroides* as well as *Arcobacter* spp. (*Epsilonproteobacteria*) and *Thiomicrospira* spp. (*Gammaproteobacteria*) (Brinkhoff et al., 1999; Sievert et al., 1999a, 2000a,b).

In this study, we carried out an environmental survey of a shallow-water hydrothermal vent located in Paleochori bay, Milos island, Greece. The site was sampled to investigate the structure and diversity of the bacterial community along a 1.5 m transect starting at the center of one of the vents.

## Materials and methods

### Site description and sample collection

The Milos hydrothermal system is one of the largest in the Mediterranean Sea. It is part of the Hellenic Arc, whose eastern section reaches the Turkish coast and the island of Kos, and Methana to the west. Extensive submarine venting occurs offshore, from the intertidal zone to depths of more than 100 m, with an approximate extension of 34 km^2^ of seabed (Dando et al., 2000).

**Figure 1:**
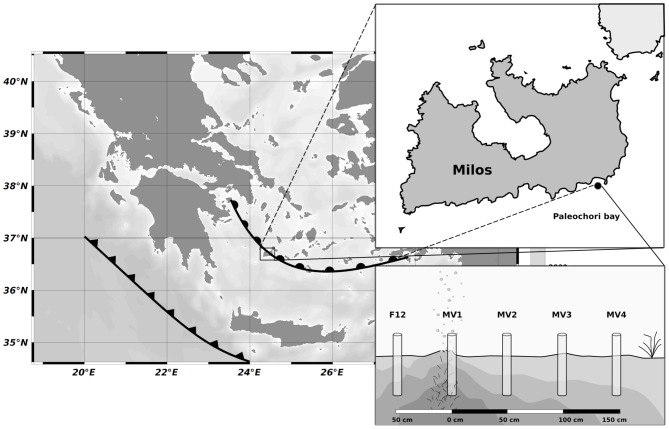
**Map showing the position of Milos (Greece) respect to the Hellenic back-arc (circles) and Hellenic Fault (triangles), the location of Paleochori Bay and the sampling strategy along the transect.** Cores were placed at interval of 50 cm moving away from the vent orifice.

Inside the Paleochori bay (Figure 1), the venting area is characterized by strong degassing activities coupled with fluid seepage (Valsami-Jones et al., 2005). The entire shallow venting area is surrounded by patches of the seagrass *Posidonia oceanica*, and the vents occur as areas of high temperature and degassing on the sandy bottom (referred to hereafter as the center of the vent) that gradually decrease to ambient conditions as the distance from the vent increases. Steep thermal and redox gradients occur vertically, with temperature increasing with the depth of the sediment, while more gradual temperature and redox changes occur horizontally as the distance from the center of the vent increases (Sievert et al., 1999a, 2000a; Dando et al., 2000; Wenzhöfer et al., 2000). Temperatures of up to 119°C at a vent site in 10 m water depth have been reported (Botz et al., 1996). Venting fluids are enriched with freshwater with varying salinity. The composition of CO_2_ released with the fluids ranged between 54.9 and 91.9%, while the concentrations of H_2_S, CH_4_ and H_2_ were ≤8.1, ≤9.7, and ≤3%, respectively (Botz et al., 1996; Dando et al., 2000). In addition, the hydrothermal fluids have been shown to contain elevated concentrations of reduced inorganic chemicals, such as NH^+^_4_ (up to 1 mM), H_2_S (up to 1 mM) and Mn^2+^ (up to 0.4 mM) (Fitzsimons et al., 1997). Arsenic and sulfur precipitates are common in proximity to vent orifices, and dense brines can be found in sediment depressions (Dando et al., 2000).

SCUBA divers collected sediment samples using push-cores during the MAMBA cruise in 2010. Starting from the center of a vent located at a depth of 12 m (designated as MV1; 36° 40.351′ N, 24° 31.108′ E), a horizontal transect consisting of four stations located 50 cm apart from each other was sampled (MV1, 0 cm distance from the center of the vent; MV2, 50 cm distance; MV3, 100 cm distance; MV4, 150 cm distance; Figure 1 and Table 1). Additionally, a single station was sampled at ca. 50 cm from the center of the vent in a yellow sediment patch (F12). The transect was characterized by a 20°C thermal gradient (45 and 25°C at stations MV1 and MV4, respectively) and differences in sediment color were observed. Station MV4 appeared only marginally influenced by hydrothermal activity as the temperature of its surface sediments was close to ambient and *P. oceanica* was observed in close proximity (Table 1).

**Table 1:** **Sampled stations, temperature, organic matter content and main characteristic of the area**.

**Station**	**Sediment depth cm**	**Surface temperature °C**	**Protein mg g^−1^**	**Carbohydrate mg g^−1^**	**Lipids mg g^−1^**	**BPC mg C g^−1^**	**CPE μg g^−1^**	**Notes**
**MV1**	0–1	45	0.70 ± 0.05	0.07 ± 0.00	0.18 ± 0.02	0.45 ± 0.03	11.84 ± 1.35	Vent orifice–dark gray sediment
	3–5		0.27 ± 0.20	0.06 ± 0.01	0.07 ± 0.00	0.19 ± 0.08	6.17 ± 3.29	
	10–15		0.39 ± 0.00	0.12 ± 0.02	0.10 ± 0.03	0.29 ± 0.03	6.12 ± 0.64	
**MV2**	0–1	32	0.53 ± 0.15	0.03 ± 0.01	0.10 ± 0.01	0.30 ± 0.06	9.31 ± 0.94	
	3–5		0.21 ± 0.03	0.02 ± 0.00	0.07 ± 0.04	0.15 ± 0.05	12.97 ± 0.64	
	10–15		0.88 ± 0.63	0.01 ± 0.00	0.14 ± 0.01	0.46 ± 0.25	2.14 ± 0.51	
**MV3**	0–1	28	0.42 ± 0.17	0.05 ± 0.01	0.21 ± 0.02	0.34 ± 0.09	25.69 ± 2.24	Presence of white precipitate
	3–5		0.10 ± 0.05	0.02 ± 0.00	0.09 ± 0.02	0.12 ± 0.01	11.37 ± 0.38	
**MV4**	0–1	25	0.20 ± 0.03	0.08 ± 0.03	0.15 ± 0.15	0.23 ± 0.08	154.6 ± 3.89	Proximity to *P. oceanica*
	3–5		0.21 ± 0.04	0.08 ± 0.02	0.24 ± 0.11	0.30 ± 0.11	72.21 ± 4.45	
	10–15		0.32 ± 0.03	0.05 ± 0.01	0.17 ± 0.08	0.28 ± 0.05	12.67 ± 0.63	
**F12**	0–1	34	3.62 ± 0.23	0.03 ± 0.00	0.06 ± 0.01	1.51 ± 0.08	7.6 ± 1.1	Yellow sediments

Means are reposted with standard deviation. BPC, Biopolymeric organic carbon; CPE, total phytopigments.

Cores were retrieved on board, sediment horizons separated (0–1, 3–5, and 10–15 cm) and processed according to the analytical procedure described in following paragraphs.

### Organic matter composition

Aliquots of sediments were immediately frozen at −20°C for determination of organic matter quantity and composition. Total protein concentrations were determined on sediment sub-samples according to Hartree (1972). Total carbohydrates were analyzed according to Gerchakov and Hatcher (1972) and expressed as glucose equivalents. Total lipids were extracted from the sediment by direct elution with chloroform:methanol (1:1 v/v) according to Bligh and Dyer (1959) and then determined according to Marsh and Weinstein (1966). All readings were performed spectrophotometrically. Carbohydrate, protein and lipid concentrations were converted into carbon equivalents using the conversion factors 0.40 and 0.49 and 0.75 mgC mg^−1^, respectively, and normalized to sediment dry weight (Fabiano et al., 1995). Biopolymeric organic carbon was calculated as the sum of the C equivalents of protein, lipid and carbohydrate.

Chlorophyll-a and phaeopigments were extracted from sediment sub-samples according to Plante-Cuny (1974). Briefly, a few mg of MgCO_3_ were added to 1 g of wet sediment to avoid chlorophyll-a degradation. Samples were supplemented with 90% acetone, sonicated and incubated in the dark at 4°C for 12 h. Following incubation, the samples were centrifuged to remove the sediment and the concentration of the pigments in the supernatant were determined spectrofluorimetrically (ex. 430 nm, em. 665 nm) before and after acidification with HCl 0.1 N. Concentrations were calculated against a standard curve and normalized to sediment dry weight. Total phytopigments (CPE) were obtained from the sum of chlorophyll-a and phaeopigments.

### Prokaryotic abundance and biomass

Total prokaryotic abundance was determined by direct counts after staining with acridine orange (Danovaro et al., 2002). Briefly, 0.5 g of each sample was supplemented with 5 mM tetrasodium pyrophosphate and incubated for 15 min before sonication. The samples were then stained with 0.025% (wt/vol) acridine orange and filtered on 0.2 mm pore-size Nucleopore black polycarbonate filters, under low vacuum (<100 mm Hg). The filters were analyzed as described by Fry (1990), using epifluorescence microscopy (Zeiss Axioskop 2; 1000 × magnification). The total prokaryotic abundance was normalized to sediment dry weight after desiccation.

Prokaryotic biovolume was estimated using the image analysis software ImageJ (Schneider et al., 2012). Average carbon content was assumed to be 310 fg C μm^3^ (Fry, 1990). The prokaryotic biomass (PBM) was normalized to sediment dry weight.

### Genomic DNA extraction and PCR amplification

Genomic DNA was extracted from sediment biomass by the phenol:chloroform method (Maniatis, 1989). Briefly, aliquots of frozen sediments (ca. 0.5 g) were resuspended in extraction buffer (100 mM Tris-HCL, 100 mM EDTA, 1.5 M NaCl pH 8.0), supplemented with 10 mg ml^−1^ lysozyme and incubated for 30′ at 37°C. Subsequently, 20% SDS was added and each sample was incubated with agitation for 1 h at 60°C. Sediments were removed by centrifugation (5 min at 14,000 × g) and supernatants were collected and extracted with 1 volume of phenol:chloroform:isoamyl alcohol (25:24:1) followed by one extraction with chloroform:isolamyl alcohol (24:1). DNA was then precipitated overnight with 0.1 volumes of sodium acetate and 0.7 volumes isopropanol, washed with 70% ice-cold ethanol, resuspended in PCR grade water and visualized on 1% agarose gel.

The bacterial 16S rRNA gene was amplified by polymerase chain reaction (PCR) using universal bacterial primer 8F (5′-AGA GTT TGA TCC TGG CTC AG-3′) and 1517R (5′-ACG GCT ACC TTG TTA CGA CTT-3′) (Weisburg et al., 1991). Aliquots of 5 μl of PCR products were visualized by staining with ethidium bromide on 1.5% agarose gel.

### Denaturing gradient gel electrophoresis

A preliminary assessment of the diversity of the sediment bacterial communities was carried out by Denaturing Gradient Gel Electrophoresis (DGGE) analysis of the bacterial 16S rRNA gene. The full-length 16S rRNA gene was obtained as described above, gel-purified and used as a template for nested PCR to amplify the V3 region using the GC-clamp primer 338F-(GC) (5′-CGC CCG CCG CGC GCG GCG GGC GGG GCG GGG GCA CGG GGG GAC TCC TAC GGG AGG CAG CAG-3′) and 519R (5′-GWA TTA CCG CGG CKG CTG-3′). DGGE was performed with a D Gene system (Bio-Rad Laboratories, Hercules, CA). PCR products (15 ml) were applied directly onto 6% (wt/vol) polyacrylamide gels in 1 × TAE (0.04 M Tris base, 0.02 M sodium acetate, 1 mM EDTA pH 7.4), with denaturant gradient from 40 to 60% (where 100% denaturant contains 7 M urea and 40% formamide). Electrophoresis was performed at a constant voltage of 45 V for 14 h. After electrophoresis, the gels were incubated for 15 min in 0.5 mg l^−1^ ethidium bromide, rinsed for 10 min in distilled water, and photographed with a UV Foto Analyst system (Fotodyne, Inc., Hartland, WI).

### Library construction and RFLP analysis

Amplified 16S rRNA gene fragments were excised from agarose gels, purified, and cloned into the pCR2-TOPO vector using the TOPO-TA Cloning Kit (Invitrogen, Inc., Carlsbad, California) following the manufacturer's instructions. The resulting ligation products were used to transform competent *Escherichia coli* TOP10 cells. Recombinant *E. coli* clones were grown on Luria-Broth media supplemented with 100 μg ml^−1^ ampicillin. Sixty to ninety clones for each library were randomly chosen and analyzed for insert-containing plasmids by direct PCR followed by gel electrophoresis of the amplified products. Insert 16S rRNA gene fragments were digested with HaeIII and MspI (Promega, Inc., Madison, Wis.) restriction endonucleases for 3 h at 37°C and subjected to Restriction Fragment Length Polymorphism (RFLP) analysis on 3% low-melting agarose gel. Clones were grouped into operational taxonomic units (OTUs) based on their RFLP profiles, and sequences (about 900 bases) were obtained from representative clones of each OTU.

### Statistical and phylogenetic analyses

Analysis of variance (ANOVA) using the statistical R-Software (R-Cran project, http://cran.r-project.org/) was carried out to identify significantly different samples, which were subjected to the Tukey HSD *post-hoc* test. Where ANOVA assumptions were rejected, a more conservative level of *p* was chosen (Underwood, 1991). DGGE profiles were analyzed with ImageJ (Schneider et al., 2012) and R-software for cluster analysis to identify significant differences in the composition of bacterial communities. Briefly, the position of each DGGE band was recorded using the ImageJ Gel plugin and the resulting matrix was fed to R for the determination of the distance matrix based on Jaccard dissimilarity and used for cluster analysis.

Sequences obtained from libraries were manually checked for quality, primers were removed and the resulting sequences were aligned using ClustalW (Larkin et al., 2007) and SeaView (Gouy et al., 2010). The software Bellerophon (Huber et al., 2004) was used to identify chimeric sequences, which were removed from the dataset (2.2 % of total sequences were identified as chimeric). Neighbor-Joining trees were constructed using the Jukes-Cantor correction and tree topologies were tested using 1000 bootstraps replications (Perriere and Gouy, 1996). Sequences of cultured bacteria retrieved as top blast hits against our sequences were included as references in the alignment. Phylogenetic designation of the sequences to a specific group was obtained by integrating blastn and EzTaxon results with phylogenetic analyses (Chun et al., 2007). Chao1 non-parametric diversity estimator and rarefaction curves were computed using Rarefaction software (http://www2.biology.ualberta.ca/jbrzusto/rarefact.php).

The sequences from this study are available through GenBank under accession numbers from KC463698 to KC463741.

## Results

### Sedimentary organic matter content

Biopolymeric organic carbon (BPC) in the surface sediments decreased along the transect from the center of the vent (MV1) toward the background station (MV4) with values ranging from 0.45 ± 0.03 to 0.23 ± 0.08 mg C g^−1^ at station MV1 and station MV4, respectively (Table 1). At station F12, BPC was highest (1.51 ± 0.08 mg C g^−1^). The observed horizontal gradients were significant (ANOVA *p* < 0.001), and a BPC minimum at a depth of 3–5 cm was observed at all stations. Proteins dominated the carbon pool at all stations with an average contribution to BPC of 55%, followed by lipids and carbohydrates. Protein contribution to the carbon pool decreased along the transect from MV1 to MV4 (Table 1). In contrast, total phytopigment (CPE) concentration increased along the transect from MV1 to MV4 (ANOVA *p* < 0.001). Chlorophyll-a concentrations were extremely low, and phaeopigments were the most abundant class. The quantity and composition of the organic matter of the yellow surface sediments at station F12 differed from that of the other stations with proteins contributing nearly 95% of the BPC (Table 1).

### Prokaryotic abundance and biomass

**Figure 2:**
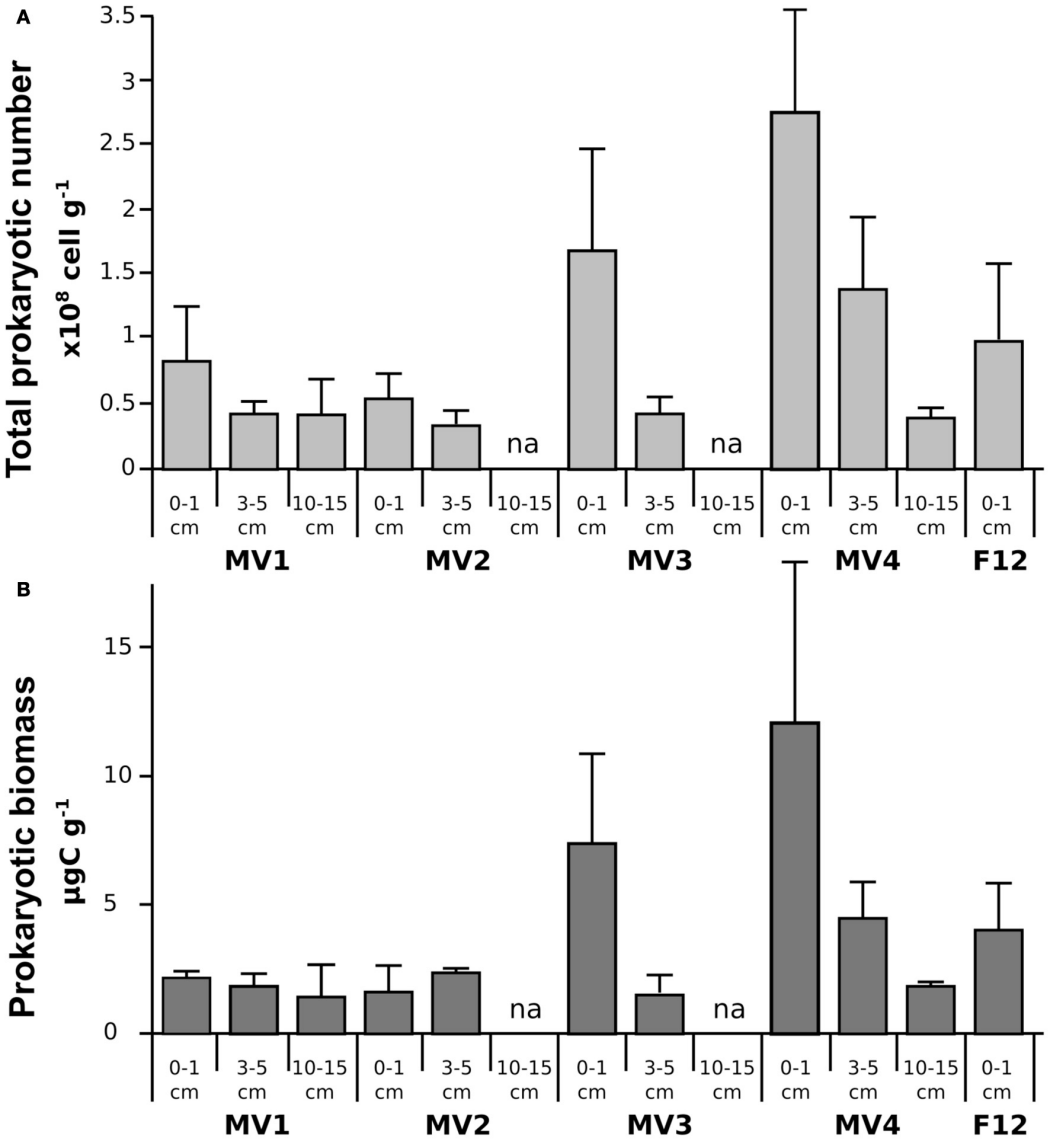
**Prokaryotic abundance (TPN; A) and biomass (PBM; B) along the sampled stations.** Mean values are reported with standard deviation. na = data not available.

In general, total prokaryotic abundance decreased as the depth of the sediment increased at all stations, despite their proximity to vent orifice (Figure 2A; ANOVA *p* < 0.001). Values ranged from 0.8 ± 0.4 × 10^8^ to 2.7 ± 1.1 × 10^8^ cells g^−1^ in the surficial sediments for MV1 and MV4, respectively, indicating higher abundances in the surface sediment layers and an increasing trend along the transect from MV1 to MV4. These trends were statistically significant (ANOVA *p* < 0.001). Prokaryotic biomass in surficial sediments ranged from 1.61 ± 1.06 to 11.95 ± 6.3 μg C g^−1^ in station MV2 and MV4, respectively (Figure 2B). Again, a trend of decreasing biomass with increasing sediment depth and increasing biomass along the transect toward the periphery of the vent was observed (Figure 2B; ANOVA *p* < 0.01). Prokaryotic abundance in station F12 was comparable to station MV1, while prokaryotic biomass was significantly higher. The decreasing trend along the vertical sediment profile was more evident at stations located further away from the center of the vent. Inter-replicate variability was also higher at those stations.

### Prokaryotic diversity

**Figure 3:**
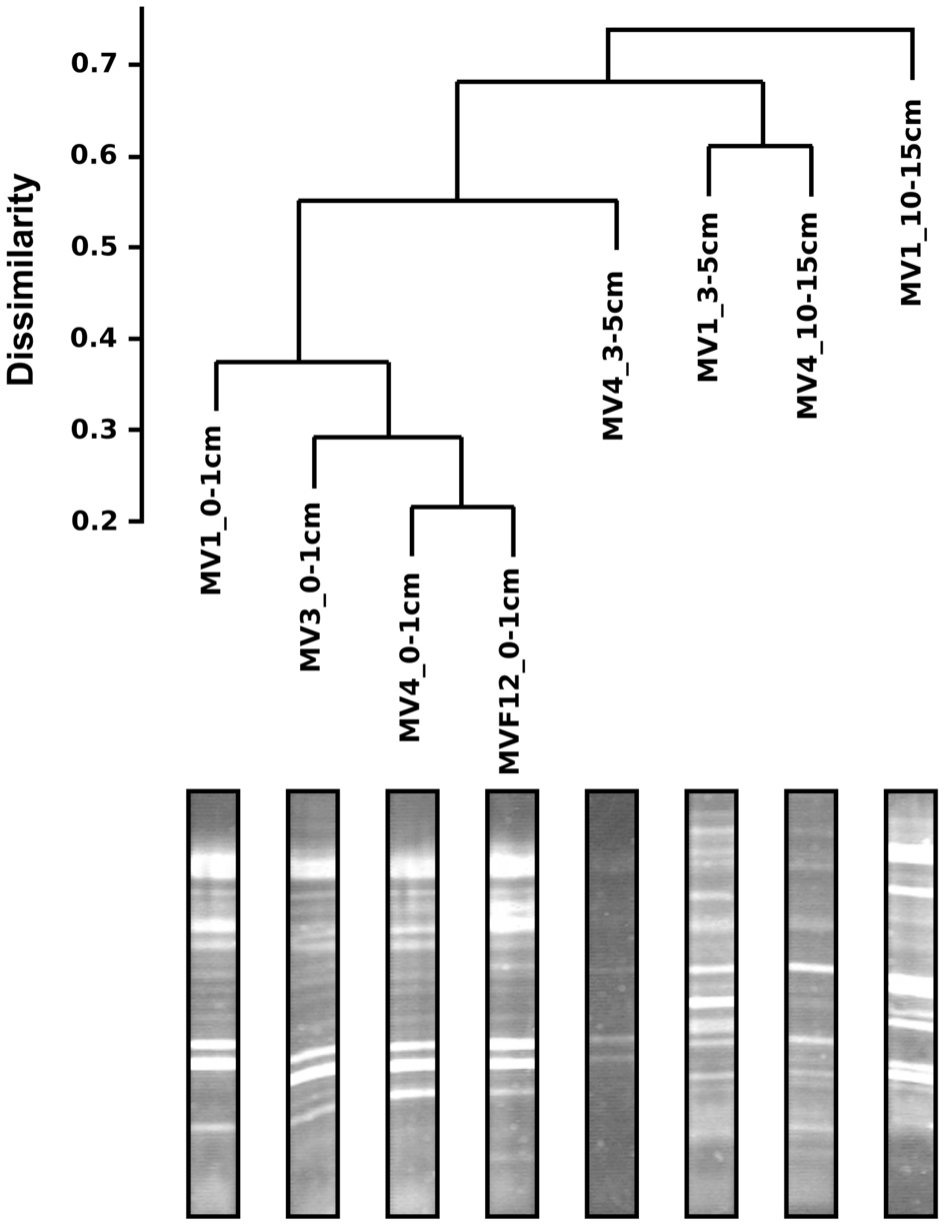
**Bacterial DGGE gel profile and cluster analysis of the resulting diversity.** Scale represents distance computed as Jaccard dissimilarity on the presence/absence matrix.

DGGE was used to investigate the diversity of the sediment bacteria from the 0–1, 5–10 and 10–15 cm layers along the transect stations, and that from the 0–1 cm layer of station F12 (Figure 3). Similar DGGE profiles were obtained from stations MV1, MV3 and MV4 and F12 (data were not obtained for station MV2). Cluster analysis based on the DGGE profiles indicated that the bacterial communities from the same sediment layers tend to group together (Figure 3). A relevant group included communities from the 0–1 cm sediments from the transect stations and from F12, while the 3–5 and 10–15 cm layers from MV1 and MV4 formed a second group (Figure 3). The bacterial communities from the 0–1 cm layer of stations MV1 (center of the vent) and MV4 (periphery of the vent) were selected to construct 16S rRNA gene libraries.

**Figure 4:**
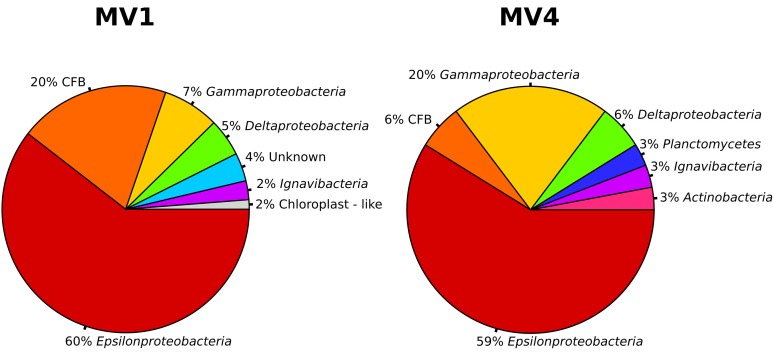
**Bacterial community structure at station MV1 and MV4 surface sediments (0–1 cm) as obtained by the analysis of the sequences.** Groups are shown at the phylum level based on the results of BLAST and position on the phylogenetic tree. *CFB, Cytophaga*-*Flavobacteria*-*Bacteroides* group.

Both the MV1 and MV4 libraries were dominated by sequences that could be assigned to the *Epsilonproteobacteria* (60 and 59% for MV1 and MV4, respectively; Figure 4). Other common phyla in both libraries were sequences belonging to the CFB (20 and 6% for MV1 and MV4, respectively; Figure 4) and *Gammaproteobacteria* (7 and 20% for MV1 and MV4, respectively; Figure 4). The number of gammaproteobacterial clones increased at the periphery of the vent and the ratio of *Epsilon*- to *Gammaproteobacteria* was 8.2 and 2.9 at stations MV1 and MV4, respectively. Sequences related to the *Deltaproteobacteria*, Planctomycetes, *Actinobacteria* and *Ignavibacteria* were also found. Four percent of the sequences retrieved from station MV1 could not be assigned to any known lineage (Figure 4).

**Figure 5:**
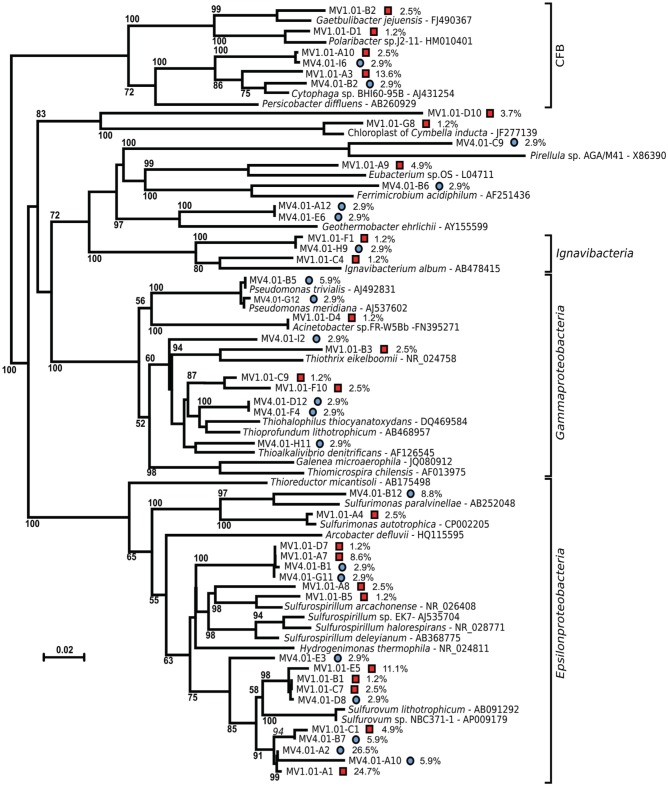
**Unrooted Neighbor-joining tree of the bacterial diversity at station MV1 (red squares) and MV4 (blue circles) surface sediments (0–1 cm) with cultured relatives.** The tree was obtained using Jukes–Cantor correction and 1000 bootstrap. Bar, 2% substitution rate. Bootstrap values below 50 are not shown. Percent values refer to the abundance of individual clones in the libraries. *CFB, Cytophaga*-*Flavobacteria*-*Bacteroides* group.

The sequences were further analyzed by aligning them against closest cultured relatives and a neighbor-joining tree was constructed (Figure 5). Both libraries are well represented in the tree, with numerous sequences clustering together despite their different origins. A large number of sequences obtained from the libraries were related to *Sulfurovum lithotrophicum* (Inagaki et al., 2004), and clustered around this sequence on the tree (average similarity of 93%; Figure 5 and Table 2). The *Sulfurovum*-related sequences were organized in two discrete clusters, each containing clones from both libraries, and represented 44% of the clones in each library. The same clusters had best hits in the non-redundant database to sequences identified during environmental surveys in a variety of submarine geothermal environments such as the Logatchev field on the Mid-Atlantic Ridge (Nakagawa et al., 2005; Hügler et al., 2010), Vailulu seamount in the Samoa and submarine volcanoes on the Kermadec Arc (Hodges and Olson, 2009).

**Table 2:** **Sequenced clones, top blast hits to the non-redundant database (closet relative) and best hit among cultured bacteria (closest cultured relative)**.

**Clone Group**	**Accession no.**	**% in library**	***Closest relative (top blast hit)***	**% similarity**	***Closest cultured relative***	**% similarity**
MV1.01-A1	KC463698	24.7	Uncultured *Epsilonproteobacterium* FN562857	92	*Sulfurovum lithotrophicum* 42BKT AB091292	94
MV1.01-A3	KC463699	13.6	Uncultured bacterium clone VS_CL-76 16S FJ497327	93	*Cytophaga* sp. BHI60-95B AJ431254	91
MV1.01-A4	KC463700	2.5	Uncultured bacterium clone VS_CL-308 16S FJ497560	95	*Sulfurimonas autotrophica* DSM 16294 CP002205	98
MV1.01-A7	KC463701	8.6	Uncultured *Epsilonproteobacterium* AB175533	90	*Sulfurospirillum* sp. EK7 AJ535704	92
MV1.01-A8	KC463702	2.5	Uncultured *Epsilonproteobacterium* AB247847	96	*Sulfurospirillum halorespirans* PCE-M2 NR_028771	90
MV1.01-A9	KC463703	4.9	Uncultured bacterium clone C13S-4 16S EU617730	95	*Eubacterium* sp. (OS type K) L04711	84
MV1.01-A10	KC463704	2.5	Uncultured bacterium partial 16S rRNA gene, SZB56 AM176864	93	*Cytophaga* sp. BHI60-95B AJ431254	90
MV1.01-B1	KC463705	1.2	Uncultured bacterium partial 16S rRNA gene AF449240	95	*Sulfurovum lithotrophicum* 42BKT AB091292	93
MV1.01-B2	KC463706	2.5	Uncultured bacterium partial 16S rRNA gene FM179896	93	*Gaetbulibacter jejuensis* CNURIC014 FJ490367	93
MV1.01-B3	KC463707	2.5	Uncultured bacterium clone MF-Oct-95 16S HQ225056	95	*Thiothrix eikelboomii* AP3 NR_024758	90
MV1.01-B5	KC463708	1.2	Uncultured *Epsilonproteobacterium* AJ969489	92	*Sulfurospirillum arcachonense* F1F6 NR_026408	95
MV1.01-C1	KC463709	4.9	Uncultured epsilon clone AT-co11 AY225616	95	*Sulfurovum lithotrophicum* 42BKT AB091292	93
MV1.01-C4	KC463710	1.2	Uncultured bacterium clone SIMO-2441 AY711807	92	*Ignavibacterium album* Mat9-16 AB478415	87
MV1.01-C7	KC463711	2.5	Uncultured Epsilonproteobacterium AB197179	93	*Sulfurovum lithotrophicum* 42BKT AB091292	94
MV1.01-C9	KC463712	1.2	Uncultured Gammaproteobacterium clone ARTE12_226 GU230337	95	*Thioalkalivibrio denitrificans* ALJD AF126545	91
MV1.01-D1	KC463713	1.2	Uncultured marine bacterium clone B-SW120 HM437670	91	*Polaribacter* sp. J2-11 HM010401	91
MV1.01-D4	KC463714	1.2	Uncultured bacterium clone ncd2696f09c JF221689	98	*Acinetobacter* sp. FR-W5Bb FN395271	98
MV1.01-D7	KC463715	1.2	Uncultured Epsilonproteobacterium AB175533	91	*Sulfurospirillum deleyianum* AB368775	88
MV1.01-D10	KC463716	3.7	Uncultured bacterium clone TF-33 16S FJ535257	94	*Thioreductor micantisoli* BKB25Ts-Y AB175498	78
MV1.01-E5	KC463717	11.1	Uncultured Epsilonproteobacterium AB197179	94	*Sulfurovum lithotrophicum* 42BKT AB091292	93
MV1.01-F1	KC463718	1.2	Uncultured bacterium clone TS-31 16S FJ535328	95	*Ignavibacterium album* Mat9-16 AB478415	88
MV1.01-F10	KC463719	2.5	Uncultured bacterium clone D13S-50 EU617758	94	*Thioalkalivibrio denitrificans* ALJD AF126545	89
MV1.01-G8	KC463720	1.2	Uncultured bacterium clone GUP5D05 16S HQ178788	94	*Cymbella inducta* NJCI77 chloroplast JF277139	94
MV4.01-A2	KC463721	26.5	Uncultured *Epsilonproteobacterium* BH8 FN562857	96	*Sulfurovum lithotrophicum* 42BKT AB091292	94
MV4.01-A10	KC463722	5.9	Epsilon ectosymbiont of Symmetromphalus GU253366	94	*Sulfurovum lithotrophicum* 42BKT AB091292	90
MV4.01-A12	KC463723	2.9	Uncultured bacterium clone SMI1-GC205-Bac66	98	*Geothermobacter ehrlichii* SS015 AY155599	88
MV4.01-B1	KC463724	2.9	Uncultured *Epsilonproteobacterium* AB175533	94	*Sulfurospirillum deleyianum* AB368775	91
MV4.01-B2	KC463725	2.9	Uncultured *Bacteroidetes* bacterium clone VS_CL-132 FJ497383	97	*Persicobacter diffluens* NBRC 15940 AB260929	84
MV4.01-B5	KC463726	5.9	Pseudomonas sp. VS05_25 FJ662886	99	*Pseudomonas trivialis* DSM 14937 AJ492831	99
MV4.01-B6	KC463727	2.9	Uncultured *Actinobacterium* AB099989	97	*Ferrimicrobium acidiphilum* T23 AF251436.2	87
MV4.01-B7	KC463728	5.9	Uncultured *Epsilonproteobacterium* FN562857	97	*Sulfurovum* sp. NBC37-1 AP009179	93
MV4.01-B12	KC463729	8.8	Uncultured bacterium clone KM51 AY216438	98	*Sulfurimonas paralvinellae* GO25 AB252048	93
MV4.01-C9	KC463730	2.9	Uncultured bacterium clone NT2_C15 HM630159	97	*Pirellula* sp. AGA/M41 X86390	87
MV4.01-D8	KC463731	2.9	Uncultured *Epsilonproteobacterium* clone R103-B76 AF449240	97	*Sulfurovum lithotrophicum* 42BKT AB091292	93
MV4.01-D12	KC463732	2.9	Uncultured sediment bacterium clone JSS S04 514 HQ191081	97	*Thiohalophilus thiocyanatoxydans* HRhD DQ469584	92
MV4.01-E3	KC463733	2.9	Uncultured *Epsilonproteobacterium* clone BSC2_c21 DQ295546	93	*Hydrogenimonas thermophila* EP1-55 NR_024811	88
MV4.01-E6	KC463734	2.9	Uncultured bacterium clone SMI1-GC205-Bac66 DQ521800	98	*Geothermobacter ehrlichii* SS015 AY155599	87
MV4.01-F4	KC463735	2.9	Uncultured sediment bacterium clone JSS S04 HQ191081	97	*Thiohalophilus thiocyanatoxydans* HRhD DQ469584	92
MV4.01-G11	KC463736	2.9	Uncultured *Epsilonproteobacterium* AB175533	95	*Sulfurospirillum deleyianum* DSM 6946 CP001816	89
MV4.01-G12	KC463737	2.9	Uncultured bacterium clone nbw124b GQ024593	99	*Pseudomonas meridiana* CMS 38 AJ537602	98
MV4.01-H9	KC463738	2.9	Uncultured bacterium clone TS-31 FJ535328	98	*Ignavibacterium album* Mat9-16 AB478415	88
MV4.01-H11	KC463739	2.9	Uncultured *Gammaproteobacterium* clone TRAN-099 JF344515	99	*Thiohalophilus thiocyanatoxydans* HRhD DQ469584	91
MV4.01-I2	KC463740	2.9	Uncultured bacterium clone SSW84Ap EU592359	97	*Thioprofundum lithotrophicum* 108 AB468957	92
MV4.01-I6	KC463741	2.9	Uncultured *Bacteroidetes* bacterium clone Belgica2005/10 DQ351801	94	*Cytophaga* sp. BHI60-95B AJ431254	90

A second major group of epsilonproteobacterial sequences was related to *Sulfurospirillum* spp. (90.8% average similarity), and clustered outside of the *Sulfurospirillum* group, constituting 13.6 and 5.8% of the two libraries respectively (Figure 5 and Table 2). Clones closely related to the *Epsilonproteobacterium Sulfurimonas authotrophica* were found exclusively in library MV1 (2.5% of the library sequences), while sequences related to *Sulfurimonas paralvinellae* constituted 8.8% of the MV4 library (93% similarity).

Sequences related to the *Gammaproteobacteria* constituted the second largest group in the MV4 library and were mainly related to *Pseudomonas* spp. and members of the *Thioalophilus*/*Thioprofundum* cluster (98.5 and 91.7% average similarity, respectively) constituting 20.6% of the clones sequenced from this library. In the library from MV1, the *Gammaproteobacteria*-related sequences were related to *Thioalkalivibrio denitrificans* and *Thiothrix eikelboomii* (average similarity 90%), constituting only 6.2% of the library. The remaining gammaproteobacterial clones were associated with *Acinetobacter sp*. FR-W5Bb (98% similarity, 1.2% of the library) and a cluster that included clones MV1.01-C9 and MV1.01-F10, related to *Thioalkalivibrio denitrificans* (90% average similarity, 3.7% of the library, Figure 5 and Table 2).

Sequences related to the *Flavobacteria*-*Bacteroides-Cytophaga* cluster were the second major group in library MV1, with an average similarity of 90% to *Cytophaga* sp. BHI60-95B (constituting 16% of the clones in the library). Sequences related to the same species were also found in library MV4 (5.9% of the library). Unique sequences related to the *Flavobacterium Gaetbulibacter jejuensis* and *Polaribacter sp*. j2-11 were present at station MV1 (93 and 91% similarity, respectively). *Deltaproteobacteria* were represented in library MV4 by sequences related to *Geothermobacter ehrlichii* (87.5% similarity, 5.8% of the clones in the library). MV1 sequences related to the *Deltaproteobacteria* represented 5% of the clones in this library and were associated to *Eubacterium* sp. OS (84% similarity).

Clones related to the *Ignavibacterium album* (87.7% similarity on average) were retrieved from both libraries, MV1 and MV4.

**Figure 6:**
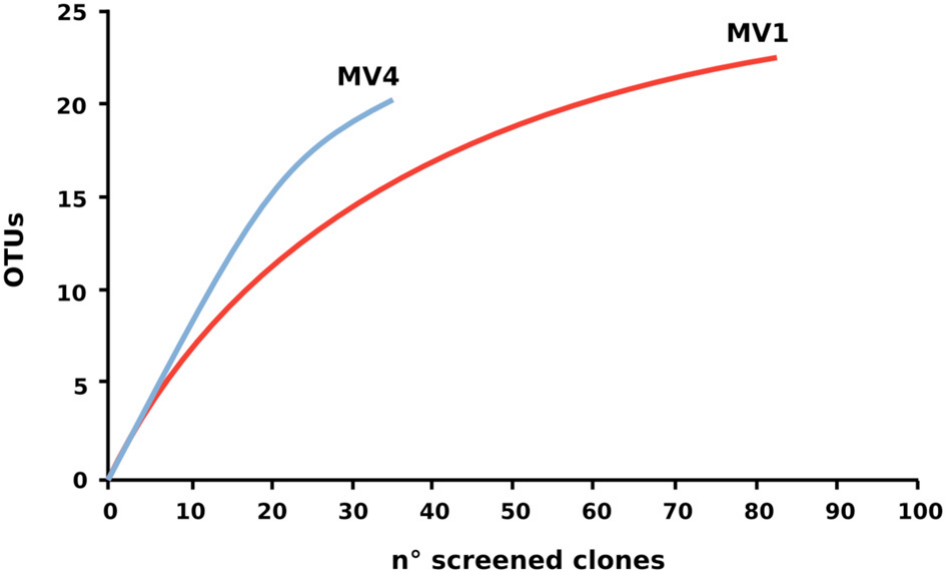
**Rarefaction curve of the 16S rRNA gene libraries.** Rarefaction curves were computed using Rarefaction software (http://www2.biology.ualberta.ca/jbrzusto/rarefact.php).

Despite the lower number of clone sequenced at station MV4, computed Chao1 diversity estimate indicated that diversity at MV4 was higher than MV1 (Chao1, 63.7 ± 22 and 28.8 ± 4.2 respectively). This can be also inferred from the slope visible on the rarefaction curves computed for both libraries (Figure 6).

## Discussion

Shallow-water hydrothermal vents are distributed worldwide and, while understudied relative to their deep-sea counterparts, they represent unique ecosystems where primary productivity is supported both by chemosynthesis and photosynthesis (Tarasov et al., 2005). In this study, we investigated the abundance, biomass, community structure and diversity of the prokaryotic community at a shallow-water hydrothermal vent in Paleochori Bay, Milos island, Greece (Figure 1).

Prokaryotic abundance was as high as 2.7 ± 1.1 × 10^8^ cells g^−1^, with average values comparable to those reported in previous studies of the same area and other shallow-water hydrothermal systems, as well as at deep-sea vents (Figure 2A; Sievert et al., 1999b, 2000a,b; Nakagawa et al., 2005; Manini et al., 2008; Williamson et al., 2008; Maugeri et al., 2009, 2010). Prokaryotic abundance decreased with depth in the sediments at all stations, a general trend previously reported for shallow-water marine sediments (Molari et al., 2012). In contrast, prokaryotic abundance increased toward the periphery of the vent (Figure 2A). Prokaryotic biomass followed similar spatial patterns, with higher values at the surface (0–1 cm) and at station MV4 (Figure 2B). A previous report of the Milos vents indicated that deeper sediments tend to be hotter (Sievert et al., 2000b), while we observed that surface sediments along the transect became gradually cooler at the periphery of the vent (Table 1). Hence, it appears that temperature and prokaryotic biomass are inversely correlated (Figure 2, Pearson moment correlation *r* = 0.617, *p* < 0.05).

Biopolymeric organic carbon (BPC), as well as proteins, decreased along the transect from the center (MV1) to the periphery of the vent (MV4), while carbohydrates and lipids remained fairly stable (Table 1). In contrast, phytopigments (CPE) followed an opposite pattern, increasing along the transect from MV1 to MV4 (Table 1). The highest concentrations of proteins and lowest concentration of CPE were measured at station F12 (yellow sediments, temperature 34°C; Table 1). The increasing concentration of CPE as the sediment temperature decreases implies an increase of phototrophic organisms in the lower temperature regions of the vent system, probably due by the presence of previously described diatoms and cyanobacteria mats in the outer rings of similar vents (Thiermann et al., 1997). This is consistent with the general notion that photoautotrophs are less tolerant to elevated temperatures than chemoautotrophic or chemoheterotrophic microorganisms (Madigan et al., 2012). These findings suggest a gradual enrichment of phototrophic organisms along the decreasing thermal gradient. While BPC and proteins decreased along the transect from MV1 to MV4, prokaryotic abundance and biomass increased (Table 1 and Figures 2A,B) and no significant correlation was found among the two parameters (Pearson moment correlation *r* = −0.214, *p* > 0.05). Since the transect was relatively short and all sampling stations were at the same depth, it is safe to assume that the input of organic matter from the water column and lateral advection was constant for all stations. We hypothesize that the decreasing prokaryotic biomass in the hottest section of the vent may lead to a decrease in carbon consumption, which is reflected in the higher concentration of measurable proteins and BPC. This hypothesis implies that temperature, rather than trophic resources, mainly controls the distribution of prokaryotes in this system.

Bacterial diversity, investigated by DGGE profiles, showed that the main DGGE banding pattern was highly conserved, while the highest number of unique bands was obtained from the 3–5 and 10–15 cm depth profiles at station MV1 (Figure 3). In total, 30 unique bands with distinct electrophoretic mobility were obtained. As expected, the DGGE analysis highlights the presence of distinct bacterial populations in surface and deeper sediments, which likely reflect different thermal and redox regimes (Figure 3). Similar findings have been reported for a variety of environments, including shallow-water and deep-sea hydrothermal vents (Moyer et al., 1995; Sievert et al., 1999b, 2000a; Manini et al., 2008).

Previous studies of the Paleochori Bay vents based on DGGE and fingerprinting analyses reported a population dominated by the *Cytophaga-Flavobacteria-Bacteroides* cluster, *Gammaproteobacteria* of the genus *Thiomicrospira* and *Epsilonproteobacteria* of the genus *Arcobacter* (Brinkhoff et al., 1999; Sievert et al., 1999a, 2000a). Here, we integrated fingerprinting, sequencing and phylogenetic analyses to assess the bacterial diversity of the shallow-water vent ecosystems of Milos island. Our survey of the bacterial 16S rRNA gene sequences of the surface sediments of stations MV1 and MV4 showed that *Epsilonproteobacteria* were the most represented class in both libraries. *Epsilonproteobacteria* are well adapted to sulfidic conditions and are commonly detected in environmental surveys of geothermal environments, as well as isolated as pure cultures from deep-sea hydrothermal vents (Campbell et al., 2006; Sievert and Vetriani, 2012). In contrast, *Gammaproteobacteria* constituted only 7 and 20% of the MV1 and MV4 clone libraries, respectively. It is worth noting that the relative abundance of gammaproteobacterial clones increased from the center to the periphery of the vent (Figure 4). The *Epsilon*- to *Gammaproteobacteria* ratio decreased accordingly, from 8.8 at station MV1 to 2.9 at station MV4. Overall, the expected diversity, calculated as Chao1 and rarefaction analyses (Figure 6), appeared to be higher at MV4. Sievert et al. (1999a) reached a similar conclusion, as they found that the microbial diversity based on DGGE profiles was higher at the periphery of the vent. Despite this, the beta-diversity within the transect appeared to be relatively low as cloned sequences retrieved from both MV1 and MV4 were closely related (Figure 5).

Phylogenetic analysis showed a large number of the sequences retrieved from both libraries were placed in the *Sulfurovum* cluster, although the average similarity of the clones generated in this study to the type strain, *Sulfurovum lithotrophicum*, was only 93% (Figure 5). *S. lithotrophicum* is a sulfur-oxidizing *Epsilonproteobacterium* isolated from deep-sea hydrothermal sediments of the Okinawa trough (Inagaki et al., 2004) and it has since been identified in deep-sea vent communities worldwide (Campbell et al., 2006; Huber et al., 2007; Tokuda et al., 2007; Huber et al., 2010). The identification of *Sulfurovum*-related clones in the Milos vents extended the distribution of this group of *Epsilonproteobacteria* to shallow-water hydrothermal systems. Currently, all the cultured *Epsilonproteobacteria* isolated from geothermal environments are chemolithoauthotrophs, although some of these bacteria have the ability to use one-carbon compounds (and in some rare cases complex organic carbon compounds; Campbell et al., 2006; Sievert and Vetriani, 2012). Given the phylogenetic distance between the *Epsilonproteobacteria* identified in this study and both cultured and uncultured relatives, it is possible that the shallow-water hydrothermal vents of Milos harbor members of the *Epsilonproteobacteria* with novel metabolic characteristics. One interesting hypothesis is that such *Epsilonproteobacteria* might be mixotrophic or facultatively heterotrophic. Herrmann et al. (2010) and Hubert et al. (2012) formulated similar hypotheses after detecting *Epsilonproteobacteria* in a benzene-degrading enrichment culture and in discharge waters collected from an oil sands reservoir, respectively. An effort to use alternative strategies to culture and isolate key members of the epsiloproteobacterial community is needed to understand their physiology and metabolism, and ultimately elucidate their role in these ecosystems.

A large number of the sequences retrieved in this study have low similarity to currently cultured bacterial strains (on average 90.1% similarity), while only four sequences (two for each library, 12.5% of the total investigated sequences) had similarities over 97%, a value considered as cut-off for closely related species/strains (Rossello-Mora and Amann, 2001). The non-redundant database indicated that the closest relatives to the Milos sequences were clones retrieved in the course of microbial diversity surveys of deep-sea hydrothermal vents (Lopez-Garcia et al., 2003; Nakagawa et al., 2005; Hügler et al., 2010), cold-seeps (Wegener et al., 2008) and seamounts (Hodges and Olson, 2009). However, the average similarity with those sequences was rather low (93.6 and 96.7% for MV1 and MV4, respectively). This was particularly true for station MV1, where the average similarity between cultured and uncultured relatives was comparable (Table 2). This suggests that the Milos shallow-water hydrothermal vents harbor previously undiscovered taxa, and raises questions about the physiology and metabolism of the *Bacteria* at this site. Aside from temperature, the quantity and quality of organic matter found at the Milos vent (Table 1) are likely a key factor in selecting for heterotrophic or facultative heterotrophic bacteria. This observation is supported by the abundance of members of the *Cytophaga-Flavobacteria-Bacteroides* (CFB) cluster in the libraries (Figure 4). Since all cultured members of the CFB are heterotrophs, it is reasonable to speculate that the Milos CFB have a similar metabolism.

None of the 16S rRNA gene sequences identified in this study were related to the newly described genus *Galenea*, isolated from the same sediment samples (Giovannelli et al., 2012). In contrast to previous studies, none of the gammaproteobacterial 16S rRNA gene sequences were related to the genus *Thiomicrospira*, and none of the epsilonproteobacterial sequences were related to the genus *Arcobacter*, both of which were previously reported to be abundant in the Milos vents (Brinkhoff et al., 1999; Sievert et al., 1999b, 2000a). This suggests that spatial and/or temporal differences in the composition of the microbial communities of these vents could be enormous. Shallow-water hydrothermal vents are characterized by fluctuating conditions with elevated temporal and spatial variability of oxygen, salinity, composition of fluids, and venting regimes (Wenzhöfer et al., 2000), and they are furthermore affected by weather conditions, swell, tides and currents. Such variability creates micro-niches and high spatial and temporal heterogeneity, possibly leading to an increase of the overall community variability and gamma-diversity.

In conclusion, we showed that prokaryotic abundances at the shallow-water vents of Milos island are comparable to those reported in other shallow-water and deep-sea hydrothermal systems. Temperature appears to be the main driving factor in controlling prokaryotic distribution in proximity of the vent and photoautotrophy seems to increase in the lower temperature regions of the vent system. For the first time, *Sulfurovum*-related sequences were found in shallow-water hydrothermal sediments, which underscores their possible relevance in the microbial communities of both shallow-water and deep-sea hydrothermal vents. The Milos shallow-water hydrothermal vent investigated in this study harbors previously undescribed and unexpected diversity, as most of the sequences retrieved had a very low similarity to previously reported ones. This may be due to the abundance of organic matter in these systems, which may support *Epsilonproteobacteria* with novel metabolic characteristics. Further attempts to isolate key species in those ecosystems will be important to shed light on their ecology and evolution and to better understand these environments.

### Conflict of interest statement

The authors declare that the research was conducted in the absence of any commercial or financial relationships that could be construed as a potential conflict of interest.
